# Non-Nutritive Sweetened Beverages Impair Therapeutic Benefits of Metformin in Prediabetic Diet-Induced Obese Mice

**DOI:** 10.3390/nu15112472

**Published:** 2023-05-25

**Authors:** Arashdeep Singh, Katelyn Rourk, Angelina Bernier, Guillaume de Lartigue

**Affiliations:** 1 Department of Pharmacodynamics, College of Pharmacy, University of Florida, Gainesville, FL 32610, USA; asingh@monell.org (A.S.); rourk.katelyn@mayo.edu (K.R.); 2 Monell Chemical Senses Center, Philadelphia, PA 19104, USA; 3 Department of Neuroscience, Perelman School of Medicine, University of Pennsylvania, Philadelphia, PA 19019, USA; 4 Mayo Clinic Alix School of Medicine, College of Medicine and Science, Rochester, MN 55905, USA; 5 Department of Pediatrics, College of Medicine, University of Florida, Gainesville, FL 32610, USA; angelina@ufl.edu

**Keywords:** artificial sweeteners, sugar, high-fat diet, diabetes, obesity

## Abstract

Metformin, a frontline therapy for type 2 diabetes and related metabolic diseases, results in variable outcomes. This study aimed to investigate whether sweetened beverages (caloric or non-caloric) affect the therapeutic benefits of metformin on glucose, food intake, and weight loss in diet-induced obesity. Mice were given a high-fat diet and sweetened water for 8 weeks to induce obesity and glucose intolerance. Then, mice were randomized to receive metformin in either water, high-fructose corn syrup (HFCS), or the non-nutritive sweetener saccharin for 6 weeks. After 6 weeks of metformin treatment, all groups had improved glucose tolerance compared to pretreatment. However, saccharin resulted in worse glucose tolerance and weight gain outcomes than the water or HFCS groups and correlated with lower plasma growth differentiation factor 15 levels. In conclusion, reducing non-nutritive sweetener consumption during metformin therapy is recommended to avoid impairing the therapeutic effects of metformin on body weight and glucose homeostasis.

## 1. Introduction

Increased consumption of western diets, characterized by calorie-dense, high-fat high-sugar foods, and sweetened beverages are considered prime suspects for the global rise in obesity and type 2 diabetes (T2D) rates [1,2]. While health agencies, including the American Heart Association, recommend a daily sugar intake of no more than 100 calories for women and 150 calories for men, the worldwide average daily sugar intake is 500 calories and is mainly attributed to the consumption of soft drinks packed with high fructose corn syrup (HFCS) [3]. Therefore, efforts aimed at reducing dietary sugars have gained attention with the goal of improving public health. Non-nutritive sweeteners (NNS), which maintain sweetness without calories, are commonly used to replace sugars. They are presumed to be inert and not to elicit a postprandial blood glucose response and prevent weight gain [4], although this remains controversial [5]. Despite the absence of strong evidence of conclusive benefits, NNS consumption has been on the rise in recent years [6]. Multiple studies conducted in rodent models have demonstrated that, like sugar, NNS increase weight gain and promote glucose intolerance through altered function and composition of gut microbiota [5,7,8]. In human studies where long-term metabolic consequences are assessed to determine the effects of NNS, metabolic features are surprisingly worse, including hypertension, dyslipidemia, diabetes, and increased cardiovascular disease risk [9,10,11].

Metformin has been the frontline treatment for T2D and for preventing or delaying the progression of diabetes in those at the highest risk for over half a century. In clinical studies, people with prediabetes and T2D take metformin to achieve improved glycemic control and moderate weight loss [12,13], although weight loss is not universal [14]. Additionally, metformin has several off-label indications as a therapy for aging, renal diseases, liver diseases, cardiovascular disease, and several cancers [15]. Epidemiological studies have shown a strong association between metformin and circulating growth differentiation factor 15 (GDF15) [16], a cellular stress metabolite expressed and secreted from multiple tissues, including the liver, gastrointestinal tract, and kidneys, that reduces food intake and body weight as well as improves glucose tolerance [17,18,19,20,21,22]. Metformin increases circulating GDF15 levels in rodents, non-human primates, and humans in a diet- and dose-dependent manner [23,24,25] and has therefore emerged as a biomarker for metformin effects. It is known that external factors, such as meal composition (fat, protein, or carbohydrates), can impact drug properties and their metabolism [26]; however, it remains unclear whether a high-fat diet and/or sweetened beverages impact the therapeutic benefits of metformin. We hypothesize that dietary sweeteners like HFCS and saccharin impair metformin-induced improvements on weight loss, normalizing glycemia and releasing GDF15 in circulation.

## 2. Materials and Methods

### 2.1. Study Design and Animals

**Figure 1:**
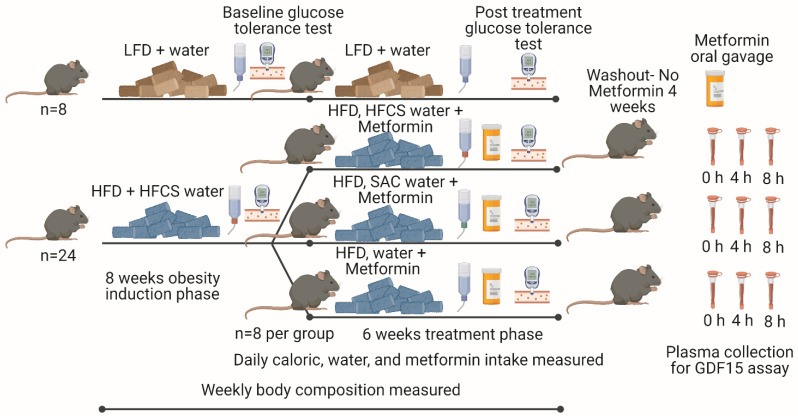
Study design to investigate the effects of consuming high-fat diet and sweetened beverages on glucose and weight homeostasis in prediabetic mice on metformin therapy. The goal of the study was to determine if sweetened drinks impair the effectiveness of metformin during the treatment phase. Our study design reflects a clinical setting in which patients present with diabetes and/or obesity and are treated irrespective of the way that these conditions developed. As part of the treatment regimen in the clinic, metformin is prescribed along with the recommendation to stop consuming sugary drinks. Therefore, to address whether the type of drink impacts metformin treatment, we provided obese/diabetic animals with metformin and the different types of drinks.

Wild-type C57BL/6J male mice (*n* = 32, 6 weeks old, Jackson Laboratories) were used in the study. Upon arrival, mice were acclimated to housing at 22–24 °C under a 12-h light-dark cycle with ad libitum access to irradiated water and a low-fat chow diet (LFD, 3.1 kcal/g, Teklad 2018, Envigo, Somerset, NJ, USA) under pathogen-free conditions in the Animal Research Facility at the University of Florida. After one week, twenty-four mice were switched to an ad libitum high-fat diet (HFD, 5.2 kcal/g, 60% calories from fat, #D12492, Research Diets, New Brunswick, NJ, USA) and sweetened water (11% *w/v* high-fructose corn syrup, HFCS) to induce obesity and a pre-diabetes state [27] (Figure 1). Eight mice were kept on LFD and water as a control group. After eight weeks, HFD-fed mice were randomized and adapted to one of three groups (*n* = 8/group): (1) continuing with HFCS, (2) water with the non-nutritive sweetener, saccharin (SAC, 0.2% *w*/*v*; matched for HFCS sweetness), or (3) water (W) (study design, Figure 1). During the first week, animals were adapted to the new solutions and fluid intake was quantified. After adaptation, the drinking water of the HFD-fed mice was spiked with metformin (MET, 300 mg/kg/day, adjusted for fluid intake) while continuing a high-fat diet for 6 weeks. All mouse experiments were performed according to the regulations and approvals of the Institutional Animal Care and Use Committee at the University of Florida, protocol number 202110305 (23 September 2021).

### 2.2. Food, Water, and Metformin Intake

Before randomizing the mice into metformin treatment groups, baseline food and water intake were measured for two consecutive days and represented as day-1. During the six-week metformin treatment phase, daily food, water, and metformin consumption were monitored using a weighing scale prior to dark onset.

### 2.3. Body Weight and Body Composition

Body weight was recorded using a conventional weigh scale and body composition was measured in awake mice by quantitative magnetic resonance using an EchoMRI™ 700 Analyzer (EchoMRI LLC, Houston, TX, USA).

### 2.4. Intraperitoneal Glucose (IPGTT) Tolerance Test

Before and after the metformin treatment phase, we performed an intraperitoneal glucose tolerance test to evaluate whether metformin and sweetened beverages differentially affect the clearance of an intraperitoneally injected glucose load from the body. Mice were acclimated to handling daily and to saline injections for 2 days before the test day to reduce the stress effects. After overnight fasting (~12 h), an IP injection of 25% glucose solution at a dose of 2 g/kg body weight was administered. Blood glucose concentrations were determined from the tail vein using a hand-held glucometer (OneTouch^®^ UltraMini^®^ glucose meter; LifeScan Inc., Malvern, PA, USA) at 0, 30, 60, and 120 min after glucose injection.

### 2.5. Plasma GDF15 Analysis

To evaluate whether sweetened beverages affect circulating GDF15 levels and whether they might impair metformin benefits, we evaluated GDF15 levels in diet-induced obese (DIO) mice following an acute metformin challenge. The test was performed after 4 weeks of post-metformin treatment. The half-life of metformin is 5–9 h [28]. Previous studies have reported a 6-day washout in rats [29], while in humans, a 2-week washout was insufficient to clear the residual effects after years of metformin treatment [30]. Based on these studies, we used a 4-week washout to minimize residual plasticity caused by a 6-week metformin treatment. During this washout period, DIO mice (*n* = 24) were discontinued on metformin but continued to receive their respective drinking solutions (HFCS, SAC, or W). For plasma GDF15 measurements, baseline plasma was collected at the end of the light cycle, after which DIO mice received gavage with metformin (300 mg/kg body weight) at the beginning of the dark cycle, and blood was collected 4 h and 8 h later from the tail vein. GDF 15 levels were measured using a commercially available rat/mouse GDF15 ELISA kit (R&D Systems, #MGD150).

### 2.6. Statistical Analysis

Statistical significance was evaluated using GraphPad Prism 9.0 software. Student’s t-test (unpaired or paired, two-tailed) was performed to compare differences between two groups. One-way ANOVA, with or without repeated measures, was used for comparing groups; two-way ANOVA, with or without repeated measures, was used for comparing more than one factor between groups, as performed for food intake, fat mass, lean mass, body weight, weight gain, fat gain, lean gain, blood glucose during IPGTT, and plasma GDF15 levels. The correction for multiple comparisons was performed using a false discovery rate at 0.05 and Benjamini, Krieger, and Yekutieli test. All data are expressed as means ± SEM and statistical significance is declared at *p* value < 0.05.

## 3. Results

### 3.1. High-Fat Diet and Sweetened Beverages Promote Obesity in Male Mice

**Figure 2:**
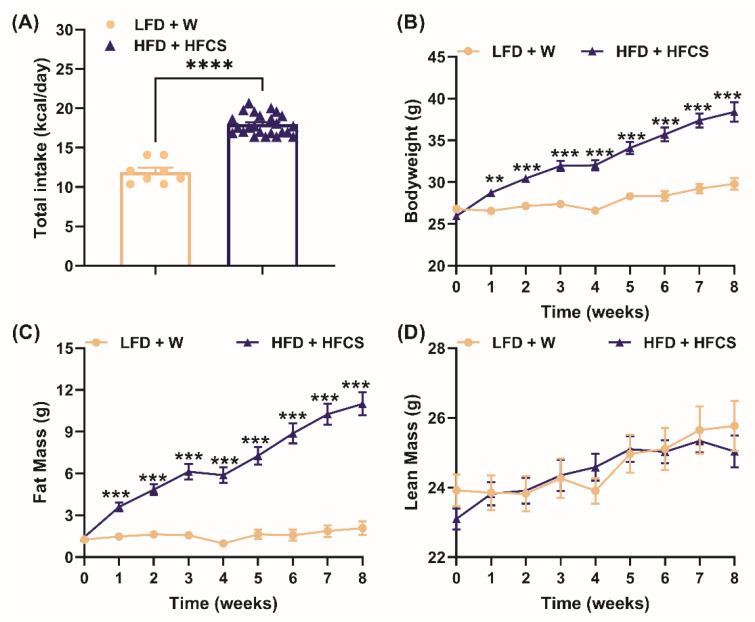
**High-fat diet and fructose-sweetened drinks increase food intake and body weight.** (**A**) Total calorie intake, (**B**) bodyweight, (**C**) fat mass, and (**D**) lean mass of male mice fed either a low-fat chow diet with water (LFD+W) (*n* = 8) or high-fat diet with 11% *w/v* high-fructose corn syrup in water (HFD+HFCS) (*n* = 24) for 8 weeks. Caloric intake was analyzed using Student’s *t*-tests (unpaired, two-sided), and body weight, fat, and lean mass were analyzed using multiple Student’s *t*-tests (unpaired, two-sided), followed by multiple comparisons correction performed using a false discovery rate at 0.5% and Benjamini, Krieger, and Yekutieli test. ns, no significance, ** *p* < 0.01, *** *p* < 0.001, **** *p* < 0.0001; LFD+W vs. HFD+HFCS.

Over the 8 weeks of obesity induction (Figure 1), HFD+HFCS mice ate more calories than LFD+W mice (*p* < 0.0001, Figure 2A). The HFD+HFCS mice weighed more (*p* < 0.0001, Figure 2B) and had greater fat mass (*p* < 0.0001, Figure 2C), but not lean mass, compared to LFD+W fed mice (*p* > 0.05, Figure 2D). Thus, as expected, the consumption of HFD and sweetened beverages promoted hyperphagia, body weight, and adiposity in mice.

### 3.2. Sweetened Beverages Attenuate Metformin-Induced Energy Balance Improvements

**Figure 3:**
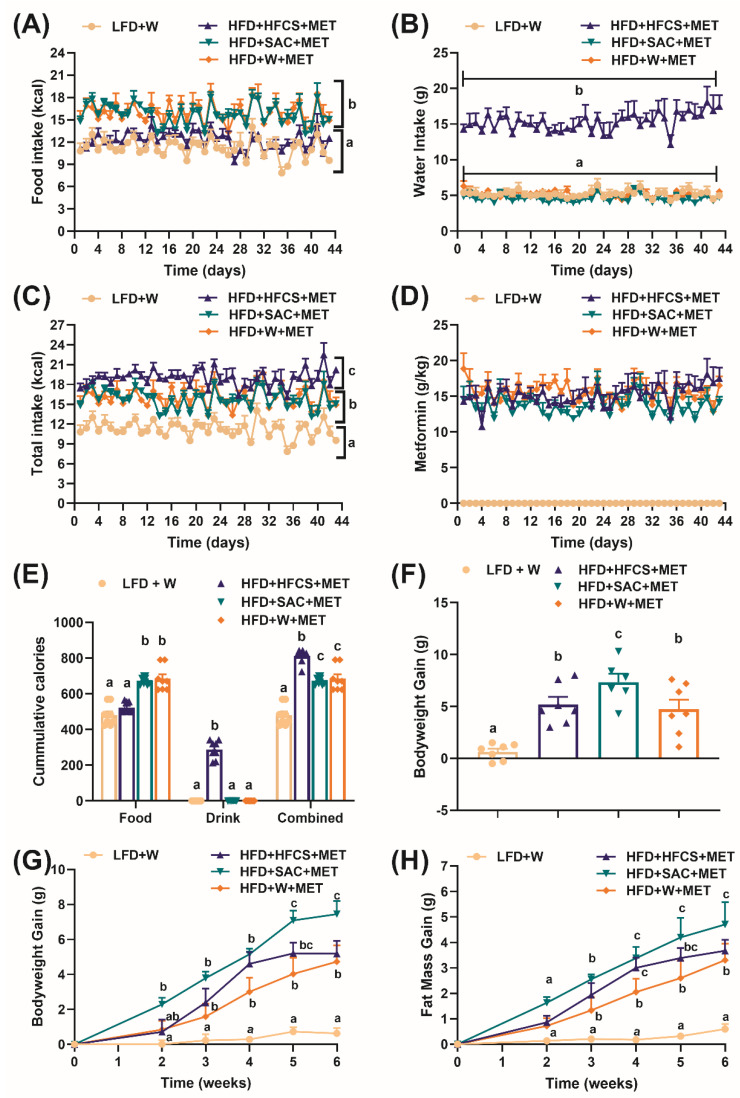
Fructose-sweetened drinks increase total caloric and water intake while saccharin sweetened drinks promote bodyweight and fat gains in diet-induced obese mice on metformin treatment. Means of daily (**A**) food intake, (**B**) water intake, (**C**) total calorie intake, and (**D**) metformin intake. Means of (**E**) cumulative calorie intake and (**F**) total bodyweight gain during 6 weeks of metformin treatment. Means of weekly (**G**) bodyweight gain and (**H**) fat mass gain. Mice (*n* = 6–7 per group) were fed either low-fat chow diet with water (LFD+W) or high-fat diet (HFD) and metformin (300 mg/kg bodyweight/day) with 11% *w/v* high-fructose corn syrup in water (HFD+HFCS+MET) or 0.2% *w/v* non-nutritive sweetener, saccharin (HFD+SAC+MET) or water (HFD+W+MET). Data presented in (**A**–**D**,**G**,**H**) was analyzed using repeated measures two-way ANOVA, followed by multiple comparisons correction performed using a false discovery rate at 0.05 and Benjamini, Krieger, and Yekutieli test. Data presented in (**E**,**F**) was analyzed using one-way ANOVA, followed by multiple comparisons correction performed using a false discovery rate at 0.05 and Benjamini, Krieger, and Yekutieli test. Values with different superscript letters ^a, b, c^ represents significant group differences (*p*  <  0.05).

Following obesity induction, DIO mice were randomized and subjected to metformin treatment (MET) with either water (W), fructose (HFCS), or saccharin-sweetened water (SAC, non-caloric) for six weeks (Figure 1). Mice were acutely acclimated to the respective solutions before starting MET treatment to avoid neophobia. Throughout the treatment period, the HFD+HFCS mice had lower food intake but higher water intake than HFD+SAC or HFD+W (*p* < 0.0001, Figure 3A,B). The dose of metformin in the drinking solution was prepared separately for each group after taking into account the different volumetric consumptions of W, HFCS, or SAC. Throughout the treatment period, the metformin dose was monitored and maintained at a concentration of 300 mg/kg body weight (Figure 3D), accounting for the higher water intake in the HFCS group (*p* < 0.0001, Figure 3B). As expected, irrespective of drinking group, the animals consuming HFD consumed more total calories per day than the control LFD+W mice (*p* < 0.0001, Figure 3C) and therefore had higher cumulative caloric intake (*p* < 0.0001, Figure 3E). Furthermore, HFD+HFCS mice consumed more total calories than HFD+SAC or HFD+W (*p* < 0.0001, Figure 3C) as a result of calories in the drinking water, which was not compensated by reduced food calories (Figure 3E).

Body weight gain and body composition analysis demonstrated that HFD-fed obese mice had a significantly higher weight and fat gain compared to LFD+W (*p* < 0.0001, Figure 3F–H). Surprisingly, despite not consuming more calories, HFD+SAC had higher weight and fat gain than HFD+W at the end of the study (*p* < 0.05, Figure 3F–H). HFD+HFCS and HFD+SAC also differed in the final week when the group with access to SAC had gained more weight and fat (*p* < 0.05, Figure 3F–H).

### 3.3. Non-Nutritive Sweetened Beverages Attenuate Metformin-Induced Glucose Improvements

**Figure 4:**
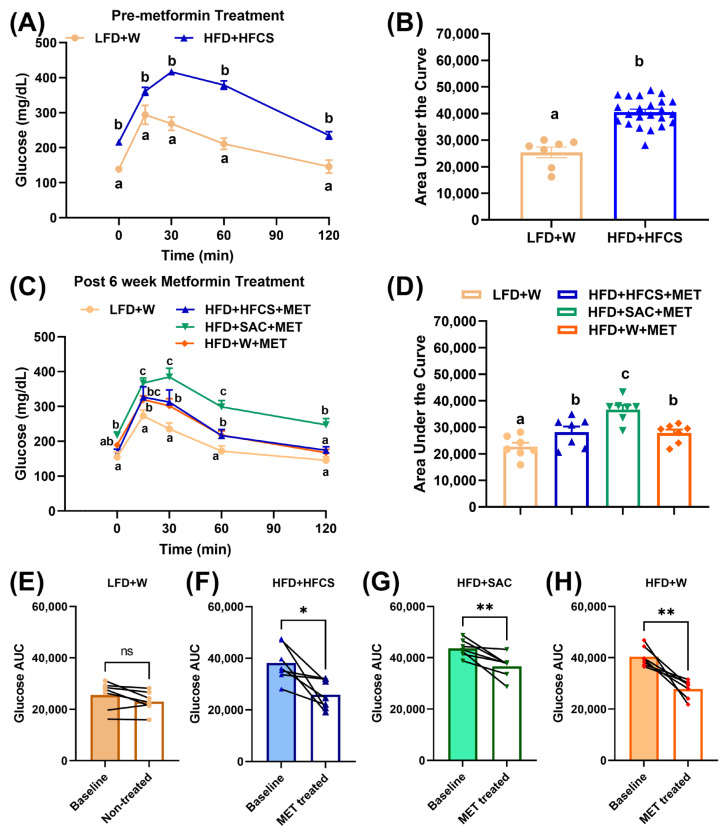
Saccharin sweetened drinks decrease improvement in glucose tolerance in diet-induced obese and diabetic mice on metformin therapy. Before metformin treatment, intraperitoneal glucose tolerance test (IPGTT) showed significant difference in (**A**) mean plasma glucose levels at baseline and 0.25, 0.5, 1, and 2 h and (**B**) area under the curve (AUC) for glucose of male mice fed either a low-fat chow diet with water (LFD+W) (*n* = 8) or high-fat diet with 11% *w/v* high-fructose corn syrup in water (HFD+HFCS) (*n* = 24) for 8 weeks. IPGTT performed 6 weeks post metformin treatment showed significant difference in (**C**) mean plasma glucose levels at baseline and 0.25, 0.5, 1, and 2 h and (**D**) AUC for glucose of mice fed either LFD+W or high-fat diet (HFD) and metformin (300 mg/kg bodyweight/day) with 11% *w/v* high-fructose corn syrup in water (HFD+HFCS+MET) or 0.2% *w/v* non-nutritive sweetener, saccharin (HFD+SAC+MET) or water (HFD+W+MET). Circulating plasma glucose levels pre- and post-metformin treatment (**A**,**C**) were analyzed using repeated measures two-way ANOVA, followed by multiple comparisons correction performed using a false discovery rate at 0.05 and Benjamini, Krieger, and Yekutieli test. AUC glucose was analyzed using one-way ANOVA, followed by multiple comparisons correction performed using a false discovery rate at 0.05 and Benjamini, Krieger, and Yekutieli test. Values with different superscript letters ^a, b, c^ represents significant group differences (*p*  <  0.05).Paired Student’s test for AUC glucose was also performed in mice on (**E**) LFD+W, (**F**) HFD+HFCS, (**G**) HFD+SAC, and (**H**) HFD+W before and after metformin treatment. ns, no significance, * *p* < 0.05, ** *p* < 0.01; baseline vs. after treatment.

At the end of the obesity induction phase, DIO mice had increased baseline circulating glucose levels and performed worse on a glucose tolerance test compared to LFD+W mice (*p* < 0.0001, Figure 4A,B), indicative of a diabetic phenotype. Specifically, when challenged with an intraperitoneal glucose tolerance test, HFD+HFCS mice displayed higher glucose levels throughout the test and thus, had a higher area under the curve (AUC) compared to LFD+W mice (*p* < 0.0001, Figure 4A,B).

We repeated the glucose tolerance test in mice after 6 weeks of metformin treatment in mice consuming either W, HFCS, or SAC to test whether sweetened beverages attenuate metformin-induced glucose improvements. Despite metformin treatment, all the HFD-fed obese mice were glucose intolerant relative to LFD+W (*p* < 0.0001, Figure 4C,D). LFD+W mice did not change their glucose clearance pattern over time (not significant, Figure 4E) while metformin improved glucose tolerance in all obese mice (*p* < 0.05, Figure 4F–H). However, HFD+SAC mice performed worse in glucose clearance after metformin treatment compared to all other groups (*p* < 0.0001, Figure 4C,D). Notably, we did not observe any glucose clearance differences between HFD+HFCS and HFD+W groups on metformin (*p* < 0.05, Figure 4C,D). Together, our results suggest that in a rodent model of diet-induced metabolic disorder, consumption of SAC impairs the beneficial effects of metformin on glucose tolerance compared to consumption of water or HFCS.

### 3.4. Non-Nutritive Sweetened Beverages Attenuate the Metformin-Induced Increase in Plasma GDF15 Levels

**Figure 5:**
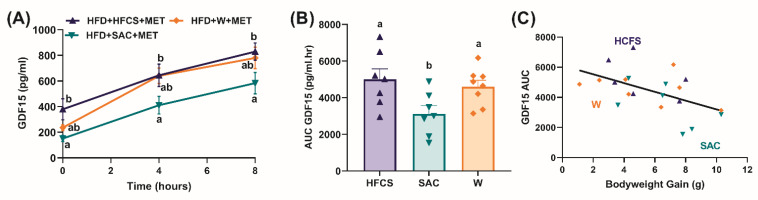
Saccharin-sweetened drinks decreased metformin-induced plasma GDF 15 levels, which correlates with higher weight gain in diet-induced obese mice. (**A**) Mean plasma growth differentiation factor 15 (GDF15) levels (*n* = 7–8/group) at baseline, 4 h, and 8 h, and (**B**) area under the curve (AUC) for GDF15 of male mice after an oral metformin gavage (300 mg/kg bodyweight). Mice were fed either a high-fat diet with 11% *w/v* high-fructose corn syrup in water (HFD+HFCS+MET) or 0.2% *w/v* non-nutritive sweetener, saccharin (HFD+SAC+MET) or water (HFD+W+MET). Circulating plasma GDF15 levels were analyzed using repeated measures two-way ANOVA (*p =* 0.02), followed by multiple comparisons correction performed using a false discovery rate at 0.05 and Benjamini, Krieger, and Yekutieli test. AUC GDF15 was analyzed using one-way ANOVA (*p =* 0.02), followed by multiple comparisons correction performed using a false discovery rate at 0.05 and Benjamini, Krieger, and Yekutieli test. Values with different superscript letters ^a, b, c^ represents significant group differences (*p*  <  0.05). (**C**) Pearson correlation between AUC GDF 15 and bodyweight gain during 6-week metformin therapy was significant (r = −0.52, *p* = 0.01).

Given the evidence that metformin increases GDF15 levels [23,24], we investigated whether sweetened beverages impaired metformin-induced GDF15 circulating levels in DIO mice. Upon completion of the 6-week metformin treatment, metformin was removed for 4 weeks to wash out chronic metformin effects and the mice were maintained on HFD and their respective water solutions. After the washout period, we performed a metformin gavage (300 mg/kg body weight) and observed that relative to both HFD+W and HFD+HFCS, HFD+SAC mice had significantly lower circulating plasma GDF15 levels (*p* < 0.0001, Figure 5A,B). We observed no differences in circulating GDF15 levels between HFD+HFCS and HFD+W groups (Figure 5A,B). We also observed a significant negative correlation between bodyweight gains and GDF15 levels independent of the group after metformin treatment (Figure 5C).

## 4. Discussion

In clinical settings, metformin is the first-line drug prescribed for T2D and has several off-label indications, including prediabetes, polycystic ovary syndrome, and prevention of weight gain when used with atypical antipsychotics. Metformin has also been demonstrated to produce moderate weight loss in several studies. Furthermore, metformin is usually prescribed along with dietary changes, including a decrease in sugar consumption. Sugary drinks are often replaced with non-caloric sweeteners to achieve lower calorie intake and weight loss. However, no study has explored the effect of nutritive or non-nutritive sweetener beverages on metformin outcomes. Here, we observed that the saccharin, a non-nutritive sweetener, did not increase caloric intake, yet it did increase body weight. Importantly, metformin-induced improvements in glucose tolerance were less pronounced in animals consuming saccharin, and this was associated with blunted GDF15 levels in circulation in response to metformin. Furthermore, we found that despite increased total caloric intake in animals consuming high-fructose corn syrup, it did not cause weight gain or increase fat mass during metformin treatment and had no negative impact on the effectiveness of metformin on improving glucose tolerance.

The consumption of sugary beverages is associated with an increased risk of diabetes and weight gain [31]. Therefore, the use of non-nutritive sweeteners has become a standard recommendation for lifestyle modification [32]. However, the substitution of sugar-sweetened beverages with artificially sweetened beverages has been shown to negatively affect multiple metabolic factors, including but not limited to increased risks for obesity and diabetes [4,33,34] and unfavorable changes in gut microbiota [5,8], despite reducing caloric intake. Additionally, interactions between diet composition and drug activity may inadvertently impair drug efficacy [26]. The Food and Drug Administration limits manufacturers to less than 12 milligrams of saccharin per fluid ounce in soda or sweet drinks and not to exceed 30 milligrams per serving size in processed foods. To translate in a preclinical setting, in this study, mice were provided with 6 milligrams of saccharin per fluid ounce, thereby limiting consumption to 3 milligrams of saccharin per day. We investigated the effects of replacing sugary beverages with non-nutritive sweeteners on caloric intake and weight gain in mice on metformin treatment. The current study aimed to closely mimic a clinical setting to address the impact of different sweetened beverages in response to metformin treatment. We found that saccharin worsened metformin outcomes, body weight, and glucose homeostasis. However, without knowing the outcome of the study prior to starting, it was not possible to test mechanisms. Potential mechanisms that should be further evaluated in future include testing the role of energy expenditure, energy absorption efficiency, microbiota, GDF15 in mediating the inhibitory effects of saccharin on metformin outcomes, and whether other NNS have the same effects.

The animals on HFCS exhibited an increase in caloric intake without any significant effect on body weight gain. This result agrees with a previous work which also reported that fructose consumption for 14 weeks increased caloric intake but did not lead to body weight gain because of a compensatory increase in energy expenditure [35]. Therefore, a compensatory increase in energy expenditure may be a potential mechanism underlying our findings. In the mice maintained on artificial sweeteners, we observed no change in caloric intake, which is consistent with a prior report [8]. Surprisingly, the mice consuming SAC gained more body weight and fat compared to those on the fructose-sweetened or regular water. The underlying mechanisms for this observation need to be further investigated, but since body weight is a balance between energy in and energy out, it is possible that alterations in energy expenditure or other metabolic adaptations, such as reduced energy absorption efficiency, may be involved. Previous studies reported that metformin can decrease food intake and prevent weight gain or promote weight loss [23,24], while others have failed to reproduce these effects [25]. Consistent with the latter study, we did not observe food intake differences before and during metformin therapy. We were not able to assess energy expenditure or energy absorption efficiency in this study, although prior work suggests that acute or chronic metformin treatment does not alter energy expenditure in HFD-fed mice [24]. The differences in the effects of metformin observed in previous studies, as well as the environmental and experimental variables in our study, highlight the need for further research to fully understand the mechanisms underlying the effects of metformin on weight loss and metabolic outcomes in different conditions. It remains possible that under certain conditions, metformin may produce moderate weight loss in mice.

High-fat diets combined with sugary beverages have been considered culprits for the increased risk of type 2 diabetes and glucose intolerance. Therefore, multiple medical practitioners and dietitians have been advising patients to replace caloric sugary drinks with zero calorie drinks that substitute sugars with artificial sweeteners for better metabolic health. However, recent landmark studies have demonstrated that chronic consumption of NNS, including saccharin, sucralose, and aspartame, exacerbates glucose intolerance compared to water, sucrose, or glucose in both chow and HFD mice by modulating the gut microbiome [8]. These deleterious metabolic effects were also conserved in humans, as demonstrated in a recent randomized control trial with 120 healthy adults [5]. Until now, it was unclear whether these sweetened beverages might be affecting the outcomes of anti-diabetes drugs like metformin. Consistent with the above findings, we observed that, while metformin improved glucose tolerance in all mice with obesity, saccharin-drinking failed to resolve glucose intolerance when compared to all other groups. These findings suggest that the current recommendations regarding NNS use during treatment should be reconsidered.

Given recent reports linking metformin therapeutic effects with GDF15 [23,24], we examined the impact of beverages on metformin-induced increases in GDF15 circulating levels. GDF15 is a newly discovered hepatocellular cytokine that is secreted from the liver, kidney, and gut in response to metformin. Metformin has been reported to increase circulating GDF15 levels in rodents, non-human primates, and humans in a diet- and dose-dependent manner, resulting in changes in food intake and glucose homeostasis [23,24,25]. Recombinant GDF15 has been reported to bind to the GDF alpha-like receptors (GFRAL) in the hindbrain to signal satiety [18,19,21,22], although there is some controversy in the field related to the role of GDF15-GFRAL signaling in mediating the feeding and body weight effects of metformin. GFRAL signaling was found to be necessary [23,24], while others suggest that the GDF15-GFRAL pathway is not critical for metformin’s effect on energy balance [25]. In this study, we found that consumption of artificially sweetened beverages decreased metformin-induced GDF15 release compared to fructose or water beverages. Additional work will be necessary to assess (1) the causal role of GDF15, (2) the endogenous levels of GDF15 required to reduce food intake, and (3) whether GDF15 control of food intake and glucose homeostasis works through different mechanisms. Previous studies have reported that metformin also improves leptin sensitivity and inhibits ghrelin in DIO rats [36,37]; therefore, it is plausible that multiple circulating factors might be involved in mediating the beneficial effects of metformin. Consumption of sweetened beverages and diabetes is prevalent in both male and female populations across different age groups; however, in this study, only young male mice were used based on previous work demonstrating that males are more prone to HFD-induced obesity and diabetes [38]. However, additional work is warranted to assess the impact on other demographics.

## 5. Conclusions

The data presented in this study challenge the current practice of substituting sugary drinks with artificially sweetened beverages for patients with obesity and diabetes who are taking anti-diabetes medications, particularly metformin. Instead, our findings suggest that artificially sweetened drinks promote obesity and diabetes and hinder the beneficial effects of anti-diabetes medications, and this is associated with decreased circulating GDF15 levels. Our preclinical findings support clinical studies on individuals with diabetes receiving metformin treatment to evaluate how beverage selection affects body weight, glycemic control, and GDF15 levels. Our findings suggest that patients undergoing treatment with anti-diabetes medications should refrain from consuming beverages with non-nutritive sweeteners.

## Data Availability

The data presented in this study are available on request from the corresponding author.

## Abbreviations

Non-nutritive sweetened (NNS); high-fructose corn syrup (HFCS); water (W); saccharin (SAC); high-fat diet (HFD); low-fat diet (LFD); type 2 diabetes (T2D); metformin (MET); growth differentiation factor 15 (GDF15); diet-induced obese (DIO); intraperitoneal glucose tolerance test (IPGTT); GDF alpha-like receptors (GFRAL).
